# In the September 2011 issue

**DOI:** 10.1590/S1980-57642011DN05030001

**Published:** 2011

**Authors:** Ricardo Nitrini

**Affiliations:** Editor-in-Chief

In this issue we are publishing a series of articles that constitute the new recommendations of the Scientific Department of Cognitive Neurology and Aging of the Brazilian Academy of Neurology (SDCNA-BAN) for the diagnosis and treatment of Alzheimer’s disease (AD) in Brazil. In 2005, recommendations were published by this same scientific department regarding the criteria to be used for the diagnosis of AD in Brazil, as well as the neuropsychological tests and questionnaires or validated scales that were more suitable for employment in Brazil, and recommendations regarding the treatment of AD.^1-3^ This year, the Alzheimer’s Association published new criteria^4^ that modify substantially the previous ones published in 1984,^5^ mainly because they include the possibility of preclinical diagnosis of AD and change the operational concept of dementia. The SDCNA-BAN adapted their recommendations to these new concepts and criteria as well as updated the items of neuropsychological evaluation and the recommendations regarding the treatment of cognitive, psychological and behavioral symptoms in AD.

In the article by **Frota et al**., it is evident that “dementia of AD” is now the substitute term for what was previously known as AD, as this disease can be diagnosed before the occurrence of any cognitive decline in its preclinical phase. The paper makes it clear that the diagnosis of preclinical AD is of interest for research and not for clinical use. The authors also stressed that the presence of memory decline is not a *sine qua non* condition for the diagnosis of dementia in AD or in other subtypes of dementia.

**Chaves et al.** carried out a systematic review of the literature to recommend tests, scales and batteries for use in the Brazilian population for the diagnosis of AD, according to the level of evidence. The authors also concluded that more validation studies should be performed.

**Caramelli et al.** evaluated, through a systematic assessment of the consensus reached in other countries and in Brazil, which are the supplementary exams that should be employed for the clinical diagnosis of AD.

**Vale et al.** carried out a systematic review to recommend the best available pharmacologic and non-pharmacologic treatments for the cognitive symptoms of the dementia of AD in Brazil. The recommendations were classified into four leves of evidence.

**Vale et al.** also evaluated the best pharmacologic and non-pharmacologic treatments for the psychological and behavioral disturbances in AD in Brazil, through a systematic review of the literature.

**Moraes et al.** compared the performance of depressive elderly, healthy older adults and healthy young in a dual-task performance. Significant differences were observed between young and old individuals.

**Kochhann et al.** evaluated the association of AD caregivers’ burden with several characteristics of the patients and concluded that neuropsychiatric symptoms were the main determinants of burden.

**Ribas et al.** compared excessive sleepiness in air traffic controllers and in control individuals using the Epworth Sleepiness Scale and the Maintenance of Wakefulness Test. Air traffic controllers exhibit excessive sleepiness.

**Ordonez et al.** compared the subjective well-being of elderly attending a third age open university for more than one semester with those starting the educational program. Participants who attended the Third Age Open University for six months or more had higher degrees of satisfaction and higher rates of psychological adjustment when compared to those beginning the university program.

**Ponce et al.** evaluated the impact of a psychoeducacional intervention on familial caregivers of AD patients using the Caregiver Burden Scale applied before and after the intervention. A positive impact of the psychoeducacional intervention was observed.

**Ricardo Nitrini** Editor-in-Chief
